# Chinese herbal medicine Xianling Gubao capsule for knee osteoarthritis: A protocol for systematic review and meta-analysis

**DOI:** 10.1097/MD.0000000000028634

**Published:** 2022-01-21

**Authors:** Wang Liu, Daoqing Xu, Qi Qi, Jingjing Li, Liang Ou

**Affiliations:** a The Second Affiliated Hospital of Guizhou University of Chinese Medicine, Guiyang 550003, China; b The Graduate School, Guizhou University of Chinese Medicine, Guiyang, 550003, China.

**Keywords:** knee osteoarthritis, protocol, systematic review, Xianling Gubao capsule

## Abstract

**Background:**

Knee osteoarthritis (KOA) is the most prevalent degenerative joint disease among populations over 60 years old, and is the most common cause of musculoskeletal pain and disability worldwide. Xianling Gubao capsule (XLGBC), a Chinese patent medicine, is widely used for treatment of osteoporosis. Meanwhile, according to the theory of homotherapy for heteropathy, XLGBC is increasingly applied in the clinical practice of KOA. However, no systematic review has found that XLGBC is as effective in treatment of KOA as it is in treatment of osteoporosis. Therefore, we will conduct a systematic review of XLGBC in KOA treatments.

**Methods:**

All randomized controlled trials assessing the validity of XLGBC therapy for KOA will be retrieved from the following seven databases, including the Cochrane Library, PubMed, EMBASE, Chinese Biomedical Literature Database, China National Knowledge Infrastructure, Wan Fang Database, and Chinese Scientific Journal Database. The primary outcome measures are the visual analogue scale pain score, and a comprehensive evaluation including the Western Ontario and McMaster Universities Arthritis Index scores, Lysholm scores, and Bristol scores. And the secondary outcome measures include cure rate and adverse events. The procedure such as retrieval and selection of literature, data extraction, evaluation of risk of bias, and assessment of reporting bias will be executed by 2 reviewers independently. The data synthesis for meta-analysis will be conducted by Review Manager 5.4 software.

**Results:**

A high-quality evidence of XLGBC for the treatment of KOA will be generated from the aspects of safety and efficacy.

**Conclusion:**

This systematic review will provide evidence to help us confirm the clinical efficacy of XLGBC in the treatment of KOA.

**OSF Registration number:**

Registration DOI 10.17605/OSF.IO/QD5SY.

## 1 Introduction

Osteoarthritis (OA) is a chronic joint disease, mainly characterized by the degeneration and destruction of the articular cartilage, the hyperostosis of the subchondral bone, and the different degrees of the synovitis.^[1]^ It is an age-related degenerative disease which seriously affects the quality of life, with high morbidity and disability among elderly people.^[2,3]^ It is reported that OA has become the fourth leading cause of disability by 2020.^[4]^ OA often occurs in the spine, hand, hip, and knee, and the knee is the most common site of symptomatic osteoarthritis. It is estimated that knee osteoarthritis (KOA) affects about 1/3 people over the age of 60, with a disability rate as high as 53%.^[5]^ Among the elderly population of China, the prevalence rate of KOA in women and men is 42.8% and 21.5% respectively, of which the prevalence rate of symptomatic KOA is 8.1%.^[6]^ In United States, the prevalence rate of symptomatic KOA is 7% to 33%.^[7]^ With the aging of social population, the incidence and disability rate of KOA will be increasing. Hence, KOA has become a major global public health problem, and the effective treatment is of great significance.

At present, there is no radical treatment available for KOA. Invariably, the main objectives in the management of KOA have been to alleviate pain, restore function, and slow down the progression of the disease. Currently, non-steroidal anti-inflammatory drugs (NSAIDs) therapy is the cornerstone of the treatment for KOA. Although NSAIDs are widely used to control the pain of osteoarthritis, many studies have found that the incidence of adverse reactions of NSAIDs is high, especially increasing the risk of gastrointestinal and cardiovascular diseases, which may threaten the patient's life and affects the quality of life for KOA patients seriously in some cases.^[8]^ In fact, researchers have recently discovered that Chinese herbal medicine may relieve the symptoms of KOA with less adverse.^[9,10]^

For thousands of years before Western medicine was introduced into China, traditional Chinese medicine, an important part of complementary and alternative medicine with complete theory, had been playing a very important role in ensuring the health of the Chinese nation. Until now, it is also the most commonly used treatment for KOA in China. Xianling Gubao capsule (XLGBC) is a Chinese herbal medicine containing 6 kinds of herbs: Epimedium Herb (Yinyanghuo, *Herba Epimedii*), Himalayan Teasel Root (Xvduan, *Radix Dipsaci*), Danshe Root (Danshen, *Radix Salviae*), Common Anemarrhena (Zhimu, *Rhizoma Anemarrhenae*), Malaytea Scurfpea Fruit (BUguzhi, *Fructus Psoraleae*), and Rehmannia Root (Dihuang, *Radix Remannieae*). According to the theory of traditional Chinese medicine, XLGBC has been widely applied for osteoporosis and OA, with the effects of nourishing liver and kidney, promoting blood circulation and communicating collaterals, and strengthening tendons and bones.^[11]^ Dialectical treatment, the same treatment for different diseases or the different treatments for same disease, is a unique concept of traditional Chinese medicine. In recent years, the clinical report of XLGBC for treatment of KOA has gradually increased. And some of the studies indicated that XLGBC was beneficial to alleviate joint pain, delay the progression of OA, and improve joint function.^[12]^ In contrast, the efficacy and safety of XLGBC in treatment of KOA has been widely debated due to the relatively small sample sizes and low quality of reporting.

So far, few researches have systematically assessed the effectiveness and safety of XLGBC treatment for KOA in accordance with the Preferred Reporting Items for Systematic reviews and Meta-Analyses (PRISMA).^[13]^ Therefore, the purpose of our study is to precisely evaluate the advantages and disadvantages of XLGBC in the treatment of KOA, and to provide evidence-based medical evidence for clinicians.

## 2 Methods

### 2.1 Study registration

**Figure 1:**
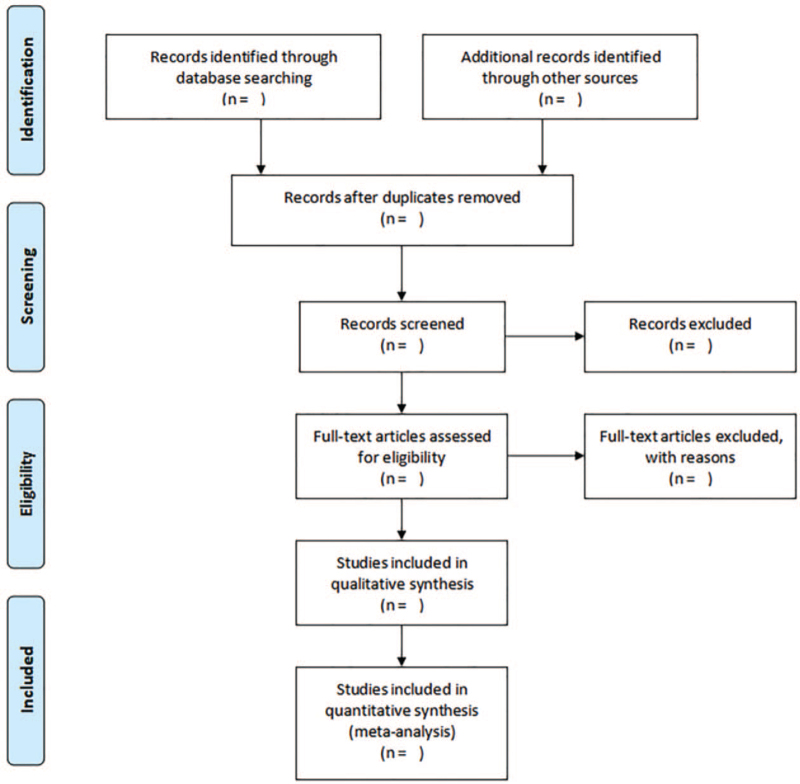
PRISMA flow diagram of study selection process. PRISMA = Preferred Reporting Items for Systematic reviews and Meta-Analyses.

The review protocol was registered on the Open Science Framework (OSF) as 10.17605/OSF.IO/QD5SY, and the protocol was reported according to Preferred Reporting Items for Systematic Reviews and Meta-Analyses Protocols (PRISMA-P) statement guidelines (Fig. 1).^[13]^

### 2.2 Inclusion criteria for study selection

#### 2.2.1 Types of studies

We will prioritize completely randomized controlled trials related to XLGBC in treatment of KOA. If not, high-quality semi-randomized controlled trials will be considered for inclusion. There are no restrictions on language and publication status.

#### 2.2.2 Types of participants

Participants with primary KOA who are diagnosed by clinical guidelines whether traditional Chinese medicine or western medicine will be selected. No age, gender, or ethnicity exemption for participants. Patients will be excluded if they have secondary KOA or other non-suppurative arthritis, such as traumatic arthritis, rheumatoid arthritis, and allergic arthritis.

#### 2.2.3 Types of interventions and controls

In randomized controlled trials, the intervention group was treated with XLGBC alone or in combination with conventional therapies. The control group included blank control, or placebo and routine treatments. There was no limit to the dosage and treatment duration between the two groups.

#### 2.2.4 Types of outcome measures

##### 2.2.4.1 Major outcomes

Improvement in joint pain and function is the main observation item, and which is respectively assessed by the visual analogue scale pain score or a comprehensive evaluation commonly using the Western Ontario and McMaster Universities Arthritis Index scores, Lysholm scores, or Bristol scores.

##### 2.2.4.2 Secondary outcomes

The secondary outcome measures include cure rate and adverse events.

### 2.3 Search methods for the identification of studies

#### 2.3.1 Electronic searches

Seven databases including Cochrane Central Register of Controlled Trials (CENTRAL), EMBASE, PubMed, China National Knowledge Infrastructure, Wanfang data, Chinese Scientific Journal Database, and Chinese Biomedical Literature Database will be searched up to November 1, 2021. We search for relevant literature in the way that a combination of subject words and free words will be used. For example, the retrieval strategy for PubMed presents as follows: (‘Xianling Gubao’ OR ‘XianlingGubao’) AND (‘osteoarthritis’ OR ‘arthritis’). The similar retrieval will be applied for the other databases.

#### 2.3.2 Other searches

In order to supplement the database searches, we will browse related academic websites comprising Google Scholar and Baidu Academic. Meanwhile, the reference lists of eligible articles and gray literature will be performed manually for obtaining more complete information.

### 2.4 Study selection and data extraction

#### 2.4.1 Selection of studies

All the relevant researches will be imported into the NoteExpress V3.2. Firstly, we remove the duplicate literature. Secondly, we directly delete those unqualified studies, such as reviews, case reports, and retrospective studies by means of reading the titles and abstracts of the remaining literature. Finally, we include the eligible literature that absolutely conform the requirements of the inclusion and exclusion criteria after reading the full text carefully. The above-mentioned work will be executed by the two researchers independently. If there is any disagreement, it will be resolved in consulting with the third researcher.

#### 2.4.2 Data extraction and management

According to predefined criteria, the two authors independently extract the following information including to the first author's name, year of publication, diagnosis criteria, sample size, sex and age of the participants, specific measures for treatment and control, follow-up, and outcome measures from each of the included studies. Discrepancies in data will be arbitrated by consensus with the third author.

### 2.5 Assessment of risk of bias in included studies

Two investigators assess the methodological quality of each trial respectively using the Cochrane collaboration's tool for assessing risk of bias. Controversy, if any, will be decided by discussion with the third party. The assessment includes the following items: random sequence generation, allocation concealment, blinding for participants and researchers, blinding for outcome assessment, integrity of outcome data, selective report, and other bias. For each study, the above seven items are judged as “low,” “unclear,” and “high.”^[14]^

### 2.6 Data synthesis and analysis

Meta-analysis will be carried out using the Review Manager 5.4 software provided by Cochrane Collaboration. In this review, continuous variables will be expressed by the standardized mean difference with 95% confidence interval, while the odds risk (OR) with 95% confidence interval, will be used for binary variables. Heterogeneity will be identified by the method of *Q* and *I*^2^ test. An *I*^2^ statistic >50% represented high heterogeneity. Subsequently, subgroup analysis or sensitivity analysis would be applied to investigate the sources of heterogeneity, when there was substantially heterogeneous. If the cause of heterogeneity still cannot be explained, we will use a random effect model to estimate the effect size. A value of *P* < .05 is considered significant.

### 2.7 Subgroup analysis

In this review, the subgroup analysis will be set in advance, and mainly consider following several aspects: intervention measures, outcome scales, treatment time, and follow-up time.

### 2.8 Sensitivity analysis

We conduct the sensitivity analysis using the method of excluding one study in each turn to detect the source of heterogeneity.

### 2.9 Assessment of reporting bias

We will conduct a funnel plot to qualitatively detect the publication bias if the number of included studies are more than 10 in this meta-analysis.

### 2.10 Grading the quality of evidence

The software of GRADEpro 3.6 will be used for grading the evidence, which is divided into high, medium, low, and very low.

### 2.11 Dealing with missing data

We will contact the author of included articles by email or telephone, when all the necessary data cannot be obtained from the literature. If it fails, then the study will be eliminated, and the potential impact of the missing data on the results will be explained further in the discussion.

### 2.12 Ethics

The ethical approval is not necessary, because our study is a secondary literature study.

## 3 Discussion

KOA is the most common form of joint disease in humans. Pain and dysfunction caused by KOA severely decreases the quality of life of patients and increases health care utilization. Currently, the etiology of KOA is still not fully understood. There is no effective cure for the treatment of KOA, and the effects of drugs which are widely used in clinic, such as NSAIDs, opioid analgesics, and sodium hyaluronate, are quite limited.^[15,16]^ Moreover, doctors and patients concern constantly about the potential adverse effects of these drugs.

At present, XLGBC is one widely used Chinese herbal medicine for patients with KOA. As far as we know, there has been no systematic review and meta-analysis on XLGBC in the treatment of KOA, although increasing studies have found that XLGBC has a statistically significant effect on alleviating symptoms of KOA with a well-established safety profile.^[17]^ Therefore, this study will be the first systematic review providing a summary of the current state of evidence regarding the safety and efficacy of XLGBC in treating KOA. We believe the results of our study will provide a new insight and reliable references for clinicians and policy makers in the treatment of KOA with XLGBC.

## Author contributions

LO is the guarantor of the article. WL and DX conceived the study idea for this study and drafted the protocol. QQ and JL developed the search strategy and contributed to the data analysis plan. LO will arbitrate any disagreement and ensure that no errors occur during the review. LO revised the manuscript. All review authors critically reviewed and approved the subsequent and final version of the protocol.

**Conceptualization:** Liang Ou.

**Data curation:** Wang Liu, Daoqing Xu.

**Formal analysis:** Liang Ou.

**Methodology:** Qi Qi.

**Software:** Jingjing Li.

**Writing – original draft:** Liang Ou, Wang Liu.

**Writing – review & editing:** Liang Ou.

**Conceptualization:** Wang Liu, Daoqing Xu.

**Data curation:** Daoqing Xu, Jingjing Li.

**Methodology:** Qi Qi, Jingjing Li.

**Project administration:** Liang Ou.

**Software:** Qi Qi.

**Supervision:** Jingjing Li.

**Writing – original draft:** Wang Liu.

**Writing – review & editing:** Daoqing Xu.
